# Blood glucose variance measured by continuous glucose monitors across the menstrual cycle

**DOI:** 10.1038/s41746-023-00884-x

**Published:** 2023-08-11

**Authors:** Georgianna Lin, Rumsha Siddiqui, Zixiong Lin, Joanna M. Blodgett, Shwetak N. Patel, Khai N. Truong, Alex Mariakakis

**Affiliations:** 1 University of Toronto, Computer Science, Toronto, ON Canada; 2 Queen’s University, Medicine, Kingston, ON Canada; 3 University College London, Institute of Sport Exercise & Health, London, UK; 4 University of Washington, Computer Science & Engineering, Seattle, WA USA

**Keywords:** Predictive markers, Reproductive signs and symptoms

## Abstract

Past studies on how blood glucose levels vary across the menstrual cycle have largely shown inconsistent results based on limited blood draws. In this study, 49 individuals wore a Dexcom G6 continuous glucose monitor and a Fitbit Sense smartwatch while measuring their menstrual hormones and self-reporting characteristics of their menstrual cycles daily. The average duration of participation was 79.3 ± 21.2 days, leading to a total of 149 cycles and 554 phases in our dataset. We use periodic restricted cubic splines to evaluate the relationship between blood glucose and the menstrual cycle, after which we assess phase-based changes in daily median glucose level and associated physiological parameters using mixed-effects models. Results indicate that daily median glucose levels increase and decrease in a biphasic pattern, with maximum levels occurring during the luteal phase and minimum levels occurring during the late-follicular phase. These trends are robust to adjustments for participant characteristics (e.g., age, BMI, weight) and self-reported menstrual experiences (e.g., food cravings, bloating, fatigue). We identify negative associations between each of daily estrogen level, step count, and low degrees of fatigue with higher median glucose levels. Conversely, we find positive associations between higher food cravings and higher median glucose levels. This study suggests that blood glucose could be an important parameter for understanding menstrual health, prompting further investigation into how the menstrual cycle influences glucose fluctuation.

## Introduction

Blood glucose levels can fluctuate due to various factors, the most well-known being body composition^1^, diet^2,3^, and exercise^4^. Understanding these fluctuations is an essential part of maintaining a healthy lifestyle as they can indicate or obscure indicators of health issues including diabetes and vascular diseases^5,6^. Individuals who menstruate are at a notably higher risk of such health issues due to hormonal imbalances, particularly those induced by life events such as pregnancy^7^ and menopause^8,9^. Although the mechanisms for these risks are unclear, periodic changes in estrogen levels during the menstrual cycle are known to play a role^10–12^. Therefore, a more robust exploration of glucose levels in non-diabetic, menstruating individuals can further our understanding of glucose variation in relation to hormonal levels and management in pathological conditions.

There is some evidence that glucose levels change across the menstrual cycle in both diabetic and non-diabetic individuals^4^, but findings across studies have been inconsistent. For instance, reports on insulin sensitivity have varied from showing a decrease during the luteal phase^13,14^ to no change across phases^15^. MacGregor et al.^4^ identified rhythmic cycling of glucose and insulin across the menstrual cycle after controlling for body mass index (BMI) and physical activity. Conversely, Yeung et al.^14^ found that glucose levels remain relatively constant throughout the menstrual cycle with slight decreases through ovulation and the earlier part of the luteal phase. With sample sizes ranging from tens to thousands of human subjects^4,13–15^, participants in these all of these studies measured their glucose levels via limited clinical blood draws, sometimes as few as one measurement per menstrual cycle phase. These studies have also often relied upon approximated or self-reported menstruation dates, which past work has found to be inaccurate for ascertaining cycle phase given strong inter- and intra-individual differences in cycle length^16,17^. Combined, these factors may contribute to the inconsistency across prior studies. To our knowledge, both continuous glucose monitors (CGM) and daily hormone-based phase predictions have not been used to study the association of glucose and the menstrual cycle in non-diabetic individuals.

In this study, we leverage CGM and daily hormone testing to examine the relationship between glucose levels and the menstrual cycle with more accurate and consistent data collection mechanisms than prior work. Furthermore, we investigate the role that potential confounders have in the cyclic nature of glucose variation across the menstrual cycle. We examine this relationship in healthy menstruating participants with no diabetic history in order to further our understanding of glucose variation without the confound of pathological conditions.

## Results

### Participant characteristics

**Fig. 1:**
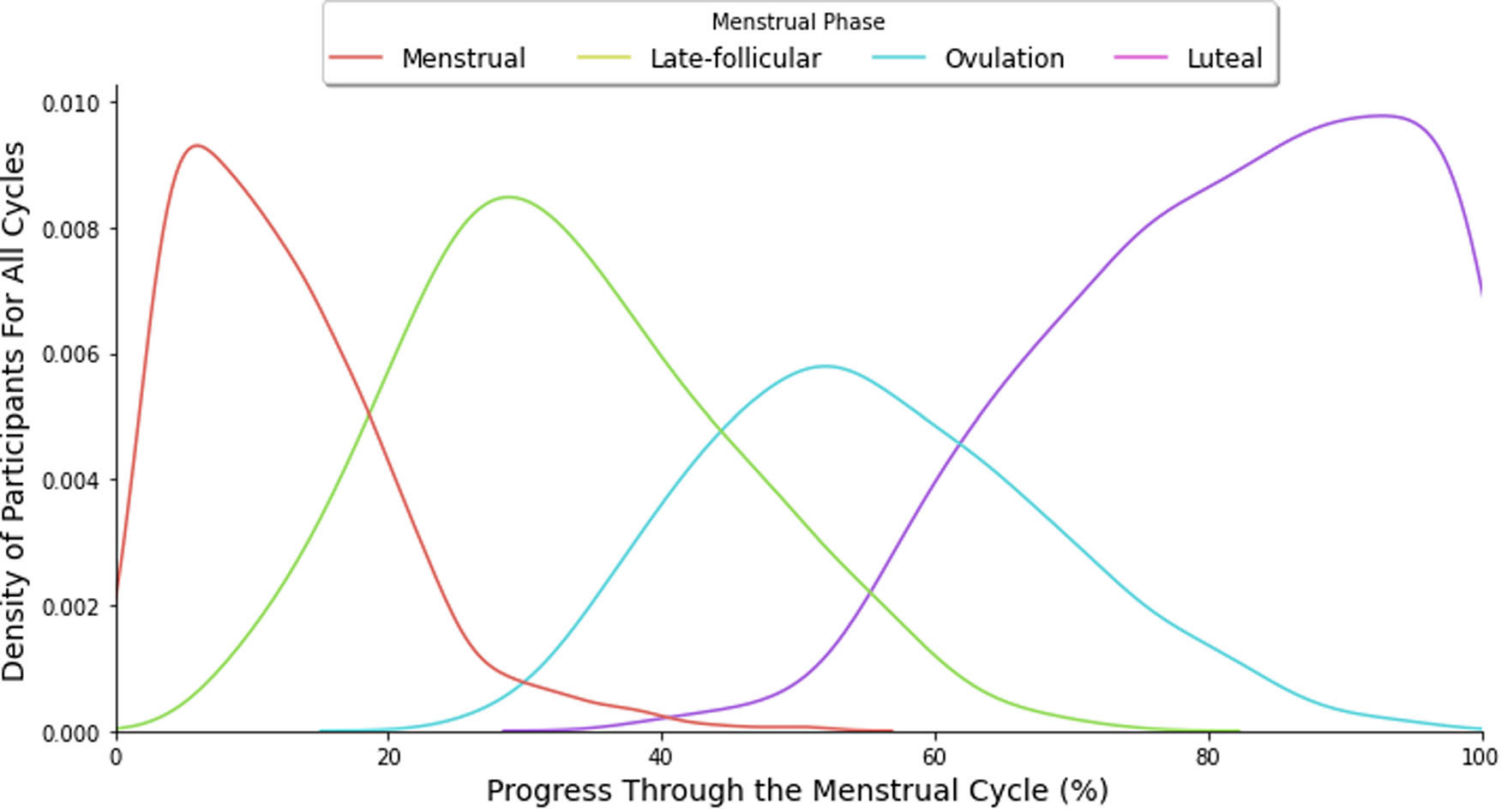
Temporal distribution of days within each menstrual cycle phase. The temporal distribution of days that were labeled with each menstrual cycle phase according to hormone data. The progression of the horizontal axis begins with the menstrual phase. The average duration of each phase across all participants was as follows: menstrual = 5.6 ± 1.6 days, late-follicular = 11.0 ± 6.1 days, ovulation = 5.9 ± 0.5 days, and luteal = 11.8 ± 3.0 days.

The characteristics of our participant cohort are summarized in Table 1 both in aggregate and separated according to menstrual cycle phase. The distribution of phase labels is illustrated in Fig. 1. According to *χ*^2^ and repeated measures ANOVA tests, we found that all of the physiological signals and self-reported experiences besides appetite level had statistically significant variation across menstrual cycle phases; detailed results of these tests can be found in Supplementary Table 1. Exercise levels and sleep issues were lowest during menstruation compared to the other phases. Conversely, food cravings, bloating, and fatigue were more prevalent during menstruation than during the other phases.

**Table 1:** Participant characteristics across menstrual phases.

	All phases	Menstrual	Late-follicular	Ovulation	Luteal
**Physiological signals (mean** **±** **standard deviation)**					
Blood Glucose (mmol/L)***	6.0 ± 0.7	6.0 ± 0.8	5.8 ± 0.7	5.9 ± 0.7	6.1 ± 0.8
Luteinizing Hormone (mIU/mL)***	5.4 ± 6.8	4.1 ± 3.2	4.7 ± 3.7	10.4 ± 12.6	3.7 ± 2.6
Estrogen (ng/mL)***	140.0 ± 112.7	104.8 ± 82.9	114.9 ± 94.2	188.6 ± 145.9	150.3 ± 105.5
Step count**	297.0 ± 213.0	290.1 ± 221.9	315.2 ± 217.3	293.1 ± 213.9	287.4 ± 203.4
**Demographics (mean** **±** **standard deviation)**					
Age (yrs)	20.8 ± 2.7				
Height (cm)	163.3 ± 5.6				
Weight (kg)	59.2 ± 11.0				
BMI (kg/m²)	22.6 ± 4.4				
**Self-reported experiences (total count of daily entries, % within menstrual phase subset)**					
Daily appetite					
Very low	164 (5%)	26 (5%)	52 (5%)	40 (6%)	46 (4%)
Low	798 (23%)	135 (24%)	231 (24%)	145 (22%)	287 (25%)
Moderate	1751 (51%)	295 (52%)	494 (51%)	342 (51%)	620 (52%)
High	597 (18%)	100 (18%)	177 (18%)	118 (18%)	202 (17%)
Very high	93 (3%)	13 (2%)	20 (2%)	23 (3%)	37 (3%)
Daily exercise level*					
Very low	698 (21%)	128 (22%)	194 (20%)	137 (21%)	239 (20%)
Low	1234 (36%)	215 (38%)	333 (34%)	241 (36%)	445 (37%)
Moderate	1079 (32%)	173 (30%)	329 (34%)	209 (31%)	368 (31%)
High	333 (10%)	39 (7%)	98 (10%)	74 (11%)	122 (10%)
Very high	57 (2%)	14 (2%)	20 (2%)	5 (1%)	18 (2%)
Daily Food Cravings**					
Did not experience	910 (27%)	111 (20%)	248 (25%)	203 (30%)	348 (29%)
Very low	620 (18%)	115 (20%)	175 (18%)	127 (19%)	203 (17%)
Low	660 (19%)	112 (20%)	196 (20%)	127 (19%)	225 (19%)
Moderate	653 (19%)	115 (20%)	190 (19%)	119 (18%)	229 (19%)
High	410 (12%)	78 (14%)	129 (13%)	73 (11%)	130 (11%)
Very high	157 (5%)	38 (7%)	39 (4%)	23 (3%)	57 (5%)
Daily bloating***					
Did not experience	1089 (32%)	116 (20%)	318 (33%)	232 (35%)	423 (35%)
Very low	693 (20%)	101 (18%)	215 (22%)	137 (20%)	240 (20%)
Low	564 (17%)	87 (15%)	188 (19%)	118 (18%)	171 (14%)
Moderate	609 (18%)	140(25%)	160 (16%)	104 (15%)	205 (17%)
High	357 (10%)	95 (17%)	84 (9%)	57 (8%)	121 (10%)
Very high	98 (3%)	30 (5%)	12 (1%)	24 (4%)	32 (3%)
Daily fatigue*					
Did not experience	410 (12%)	56 (10%)	109 (11%)	97 (14%)	148 (11%)
Very low	482 (14%)	84 (15%)	145 (15%)	90 (13%)	163 (14%)
Low	571 (17%)	91 (16%)	163 (17%)	112 (17%)	205 (17%)
Moderate	981 (29%)	154 (27%)	275 (28%)	174 (26%)	378 (32%)
High	706 (21%)	134 (24%)	216 (22%)	136 (20%)	220 (19%)
Very high	260 (8%)	50 (9%)	69 (7%)	63 (9%)	78 (7%)
Daily sleep issues*					
Did not experience	606 (18%)	81 (14%)	160 (16%)	132 (20%)	233 (20%)
Very low	664 (19%)	112 (20%)	219 (22%)	105 (16%)	228 (19%)
Low	803 (24%)	146 (26%)	231 (24%)	155 (23%)	271 (23%)
Moderate	737 (22%)	118 (21%)	210 (21%)	148 (22%)	261 (22%)
High	382 (11%)	76 (13%)	102 (10%)	74 (11%)	130 (11%)
Very high	218 (6%)	36 (6%)	55 (6%)	58 (9%)	69 (6%)

Participant demographics, physiological signals, and self-reported symptoms reported in aggregate and separated according to menstrual phase. Significant results (*p < 0.05, **p < 0.01, ***p < 0.001) of *χ*^2^ and repeated measures ANOVA tests of symptom variance across cycle phases are also indicated.

### Modeling glucose levels across the menstrual cycle

**Fig. 2:**
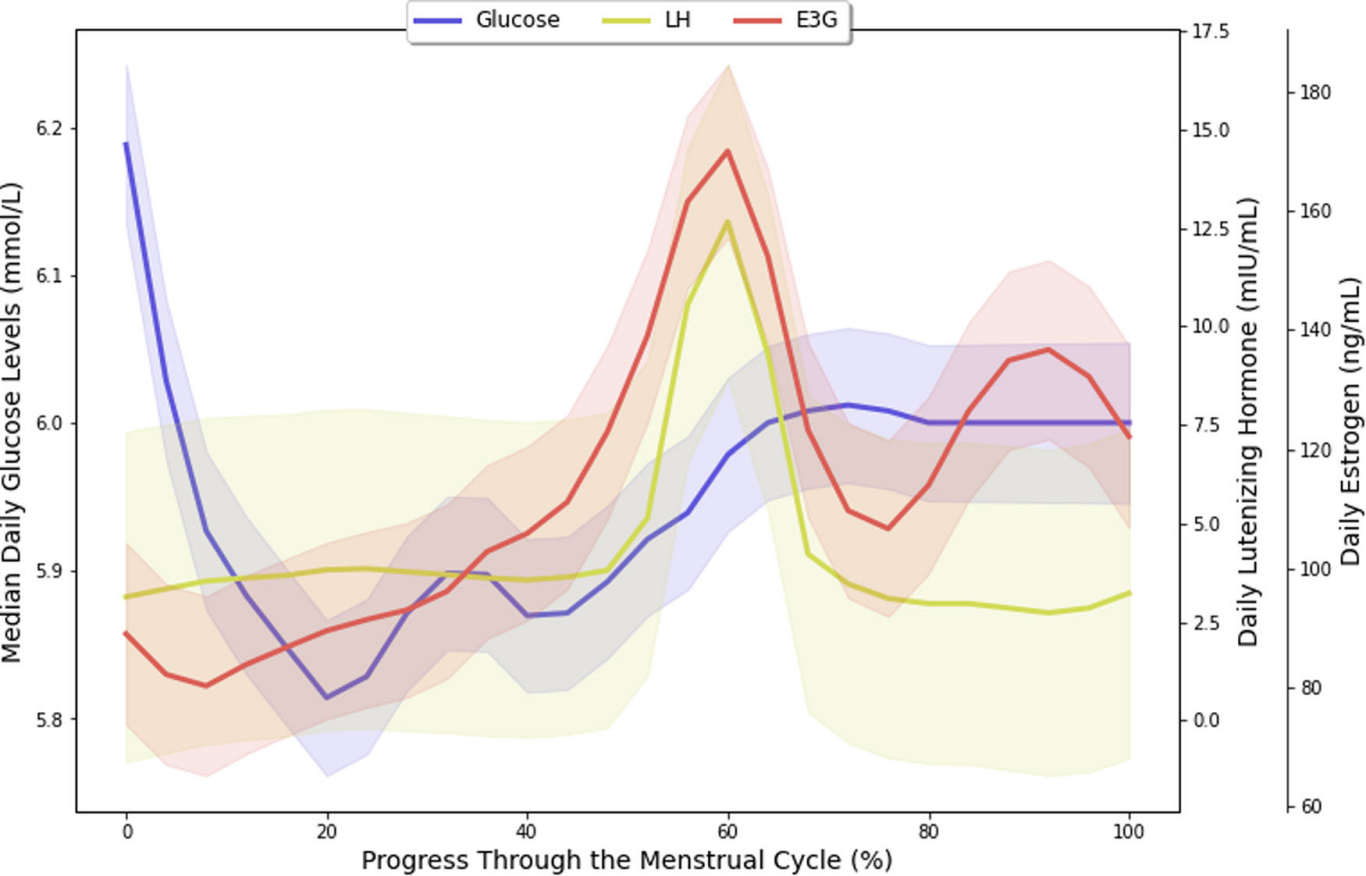
Plot of daily median glucose, estrogen, and luteinizing hormone levels throughout the menstrual cycle. A plot of daily median glucose, estrogen, and luteinizing hormone levels throughout the menstrual cycle starting from menstruation with LOESS smoothing. The shaded regions indicate 95% confidence intervals.

**Fig. 3:**
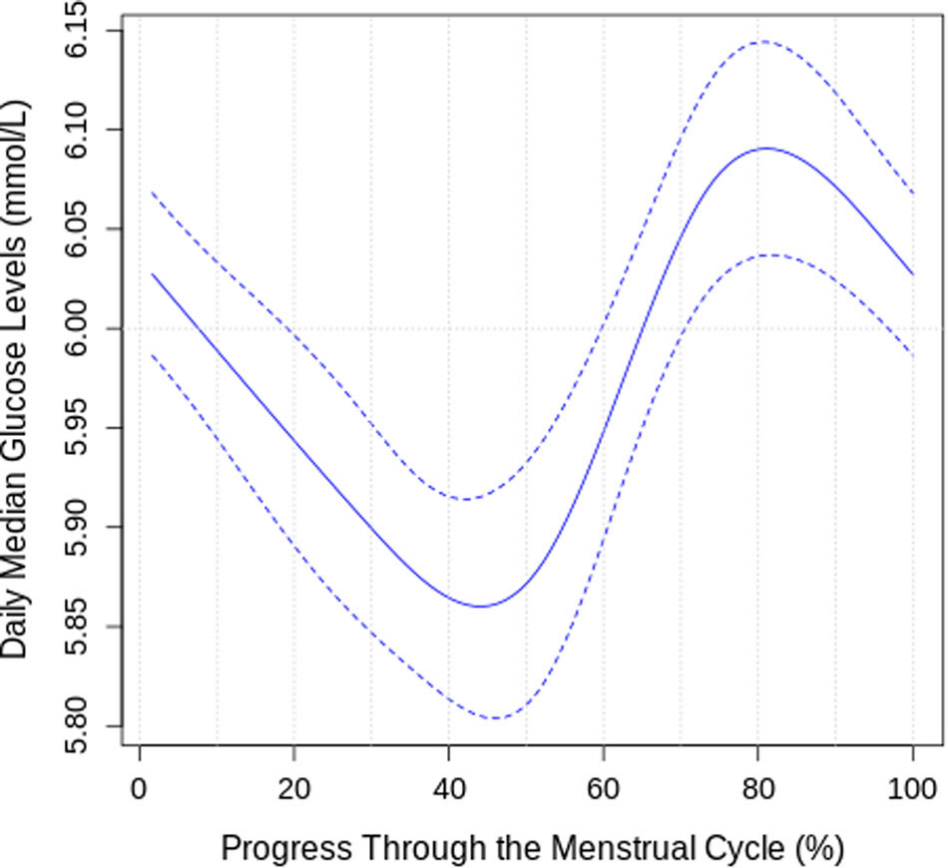
Periodic restricted cycle spline fit of daily median glucose levels changes throughout the menstrual cycle. A plot of daily median glucose levels changes throughout the menstrual cycle starting from menstruation with a periodic restricted cyclic spline fit.

Figure 2 shows the trends of daily median glucose, estrogen, and luteinizing hormone levels across the entire menstrual cycle. Figure 3 shows the trend of daily median glucose level after a restricted cubic spline analysis, and Supplementary Fig. 1 shows the distribution of these values split according to discrete menstrual cycle phases. Glucose levels were lowest during the late-follicular phase (5.8 ± 0.7 mmol/L), gradually increased during ovulation, peaked during the luteal phase (6.1 ± 0.8 mmol/L), and dropped sharply during menstruation. Where Day 1 was considered the first day of menstruation, the lowest median glucose level typically occurred at Day 13.6 ± 3.4 (late-follicular phase), and the highest median glucose level occurred at Day 24.5 ± 8.0 (luteal phase).

### Univariate associations between glucose levels, menstrual cycle phase, and confounds

As shown in Table 2, linear mixed-effects models indicated a significant increase in glucose levels during the ovulation (*β* = 0.06, *p* < 0.05, 95% CI [0.001, 0.12]), luteal (*β* = 0.15, *p* < 0.001, 95% CI [0.09, 0.20]), and menstrual (*β* = 0.09, *p* < 0.01, 95% CI [0.02, 0.16]) phases relative to the late-follicular phase. There was also a statistically significant relationship between estrogen and glucose, with higher estrogen levels leading to lower daily median glucose levels (*β* = −0.0003, *p* < 0.05, 95% CI [–0.001, –0.000001]).

**Table 2:** Univariate and multivariate linear mixed-effects models results.

	Covariate	Univariate Model	Multivariate Model
		β-coefficient [95% CI]	β-coefficient [95% CI]
**Physiological signals**			
Menstrual phase	Late-follicular	Reference	Reference
	Ovulation	0.06 [0.001, 0.12]*	0.08 [0.01, 0.15]*
	Luteal	0.15 [0.09, 0.20]***	0.16 [0.10, 0.22]***
	Menstrual	0.09 [0.02, 0.16]**	0.03 [-0.04, 0.11]
Others	Luteinizing Hormone (LH)	–0.001 [-0.01, 0.003]	–
	Estrogen (E3G)	–0.0003 [–0.001, –0.00001]*	–0.0003 [–0.001, –0.0001]**
	Step Count	–0.0003 [–0.0004, –0.0002]***	–0.0003 [–0.0004, –0.0002]***
**Demographics**			
	Age (yrs)	0.01 [–0.04, 0.05]	–
	Height (cm)	–0.01 [–0.04, 0.02]	–
	Weight (kg)	0.002 [–0.01, 0.02]	–
	BMI (kg/m²)	0.01 [–0.03, 0.05]	–
**Self-reported experiences**			
Daily appetite	Very low	Reference	Reference
	Low	–0.02 [–0.13, 0.10]	–
	Moderate	0.04 [–0.08, 0.15]	–
	High	–0.01 [–0.13, 0.12]	–
	Very high	–0.02 [–0.20, 0.15]	–
Daily exercise	Very low	Reference	Reference
	Low	–0.02 [–0.08, 0.05]	–
	Moderate	–0.02 [–0.09, 0.05]	–
	High	–0.08 [–0.17, 0.02]	–
	Very high	–0.05 [–0.24, 0.13]	–
Daily food cravings	Did not experience	Reference	Reference
	Very low	0.05 [–0.05, 0.14]	0.05 [–0.05, 0.15]
	Low	0.15 [0.07, 0.24]***	0.19 [0.10, 0.28]***
	Moderate	0.19 [0.10, 0.28]***	0.21 [0.12, 0.30]***
	High	0.15 [0.06, 0.24]**	0.17 [0.07, 0.27]***
	Very high	0.08 [–0.05, 0.20]	0.04 [–0.09, 0.17]
Daily bloating	Did not experience	Reference	Reference
	Very low	–0.06 [–0.15, 0.03]	–
	Low	0.02 [–0.06, 0.11]	–
	Moderate	0.04 [–0.05, 0.13]	–
	High	0.07 [–0.03, 0.17]	–
	Very high	–0.07 [–0.22, 0.08]	–
Daily fatigue	Did not experience	Reference	Reference
	Very low	–0.14 [–0.26, −0.04]**	–0.16 [–0.28, –0.03]*
	Low	0.03 [–0.07, 0.14]	0.02 [–0.09, 0.13]
	Moderate	0.01 [–0.09, 0.10]	–0.03 [–0.13, 0.08]
	High	0.03 [–0.07, 0.13]	–0.03 [–0.14, 0.08]
	Very high	0.08 [–0.03, 0.20]	0.03 [–0.10, 0.16]
Daily sleep issues	Did not experience	Reference	Reference
	Very low	0.001 [–0.10, 0.10]	0.05 [–0.06, 0.16]
	Low	–0.04 [–0.13, 0.06]	–0.06 [–0.16, 0.05]
	Moderate	0.04 [–0.06, 0.13]	0.02 [–0.09, 0.13]
	High	0.11 [0.004, 0.22]*	0.05 [–0.07, 0.17]
	Very high	0.09 [–0.03, 0.22]	0.02 [–0.12, 0.16]

The results of the univariate and multivariate linear mixed-effects models that were generated to estimate changes in median blood glucose across menstrual phases. Significant model results are indicated as such *p < 0.05, **p < 0.01, ***p < 0.001.

Higher daily step counts were associated with statistically significant decreases in daily median glucose levels (*β* = −0.0003, *p* < 0.001, 95% CI [–0.0004, –0.0002]). Food cravings were often associated with increases in daily median glucose levels ("low": *β* = 0.15, *p* < 0.001, 95% CI [0.07, 0.24]); "moderate": *β* = 0.19, *p* < 0.001, 95% CI [0.10, 0.28]; "high" (*β* = 0.15, *p* < 0.01, 95% CI [0.06, 0.24]). Meanwhile, there was some evidence to suggest that fatigue was significantly associated with decreasing daily median glucose levels ("very low": *β* = −0.14, *p* < 0.01, 95% CI [–0.26, –0.04]). Finally, there was an association between higher levels of self-reported sleep issues and elevated daily median glucose levels ("high": *β* = 0.11, *p* < 0.05, 95% CI [0.004, 0.22]; "very high": *β* = 0.09, *p* = 0.13, 95% CI [–0.03, 0.22]). Participants’ recorded demographics and self-reported appetite, exercise, and bloating levels did not demonstrate any significant trends with daily median glucose levels.

### Multivariate association between glucose levels, menstrual cycle phase, and confounders

For the multivariate linear mixed-effects model presented in Table 2, we included all of the variables that had significant relationships with daily median glucose levels: estrogen, step count, food cravings, fatigue, and sleep issues. The model confirmed that the association between menstrual cycle phase and median glucose level is robust to confound adjustment. Similar to the corresponding univariate model, daily median glucose levels were elevated during the ovulation (*β* = 0.08, *p* < 0.05, 95% CI [0.01, 0.15]) and luteal (*β* = 0.16, *p* < 0.001, 95% CI [0.10, 0.22]) phases relative to the late-follicular phase. However, the same could no longer be said for glucose levels during menstruation since the relationship lost statistical significance. Increased estrogen also led to decreases in daily median glucose levels (*β* = −0.0003, *p* < 0.01, 95% CI [–0.001, –0.0001]).

Associations between confounders and median glucose levels largely did not change. Higher daily step counts were associated with statistically significant decreases in daily median glucose levels (*β* = −0.0003, *p* < 0.001, 95% CI [–0.0004, –0.0002]). Food cravings were associated with increased daily median glucose levels, particularly when being reported as "low" (*β* = 0.19, *p* < 0.001, 95% CI [0.10, 0.28]), "moderate" (*β* = 0.21, *p* < 0.001, 95% CI [0.12, 0.30]), and "high" (*β* = 0.17, *p* < 0.001, 95% CI [0.07, 0.27]). As before, fatigue was only found to be significantly associated with daily median glucose levels at "very low" levels (*β* = −0.16, *p* < 0.05, 95% CI [–0.28, –0.03]). Reported sleep issues were not significantly associated with daily median glucose levels after adjustment for confounders.

We examined interactions between each of the aforementioned confounders and menstrual cycle phase. There were no significant interactions between estrogen, step count, or sleep issues with phase (all *p* > 0.05). As there were significant interactions for food cravings and fatigue with menstrual cycle phase, we stratified the data by phase and generated univariate models for each confounder. The results of this analysis are shown in Table 3. There were positive associations between higher food cravings and daily median glucose levels during the ovulation and luteal phases. There was some association between the two during the menstrual phase, albeit only on days with "very high" food cravings. Any experience of fatigue was associated with higher glucose levels during the menstrual phase, with no associations during the late-follicular or ovulation phases. The opposite pattern emerged during the luteal phase, during which those with "very low" fatigue had lower glucose levels than those who did not experience any fatigue. Associations for both food cravings and fatigue remained the same in phase-stratified multivariate models with all significant confounders included.

**Table 3:** Univariate linear mixed-effects models stratified by menstrual cycle phase results.

	Covariate	Menstrual phase	Late-follicular	Ovulation phase	Luteal phase
**Self-reported experiences**					
Daily Food Cravings	Did not experience	Reference	Reference	Reference	Reference
	Very low	0.14 [–0.11, 0.39]	–0.10 [–0.26, 0.07]	0.07 [–0.12, 0.26]	–0.003 [–0.16, 0.15]
	Low	0.12 [–0.11, 0.36]	–0.05 [–0.20, 0.10]	0.21 [0.03, 0.39]*	0.14 [0.003, 0.28]*
	Moderate	0.09 [–0.13, 0.32]	0.04 [–0.11, 0.20]	0.19 [0.01, 0.36]*	0.25 [0.11, 0.39]***
	High	0.11 [–0.12, 0.34]	-0.03 [–0.19, 0.14]	0.25 [0.06, 0.45]*	0.17 [0.01, 0.32]*
	Very high	0.34 [0.07, 0.61]*	–0.15 [–0.40, 0.09]	0.18 [–0.11, 0.47]	0.11 [-0.10, 0.31]
Daily Fatigue	Did not experience	Reference	Reference	Reference	Reference
	Very low	0.25 [–0.01, 0.50]	–0.06 [–0.26, 0.13]	–0.13 [–0.37, 0.12]	–0.30 [–0.50, -0.10]**
	Low	0.34 [0.10, 0.57]**	0.10 [–0.08, 0.28]	–0.08 [–0.30, 0.14]	–0.11 [–0.28, 0.07]
	Moderate	0.30 [0.08, 0.52]**	–0.02 [–0.19, 0.15]	–0.08 [–0.29, 0.13]	–0.04 [–0.21, 0.13]
	High	0.26 [0.02, 0.49]*	–0.03 [–0.21, 0.15]	–0.08 [–0.30, 0.13]	0.03 [–0.15, 0.21]
	Very high	0.31 [0.05, 0.58]*	0.14 [–0.07, 0.36]	–0.02 [–0.26, 0.23]	–0.06 [–0.28, 0.16]

The results of the univariate linear mixed-effects models stratified by menstrual cycle phase for confounders that resulted in statistically significant (*p < 0.05, **p < 0.01, ***p < 0.001) interactions.

## Discussion

In this study, we identified significant associations between blood glucose and the individuals’ menstrual cycles. We observed a biphasic pattern across the menstrual cycle where daily median glucose levels peaked during the luteal phase and declined during the late-follicular phase. The increase in glucose levels from the late-follicular phase to the luteal phase was also robust to a number of confounders, including step count, estrogen, food cravings, fatigue, and sleep issues.

Our findings align with studies on the physiological relationships between metabolism and hormones associated with the menstrual cycle. Estrogen promotes lipolytic action, thereby increasing triglyceride levels and inhibiting food intake^18^. Estrogen also suppresses food cravings by stimulating anorexigenic and satiety neurons in the central nervous system^19^. Our analyses reflect this relationship, as we observed strong positive associations between higher levels of food cravings and daily median blood glucose levels during the ovulation and luteal phases. Since estrogen levels are on average lowest during menstruation and highest during ovulation, the aforementioned physiological mechanisms could explain the differences we observed in blood glucose levels and food cravings across menstrual cycle phases.

Still, our findings share commonalities and differences with past literature on insulin resistance in the menstrual cycle. The most similar findings were from MacGregor et al.^4^, who uncovered rhythmic blood glucose changes across the menstrual cycle, and from Dey et al.^1^, who found that glucose peaks during the luteal phase. Our methodology differed from these works by characterizing daily glucose levels via continuous blood glucose monitoring. Unlike blood draws conducted in labs, CGMs are more cost-effective^20,21^ and enable data collection within and across days^22^. We were therefore able to examine the robustness of these associations to inter- and intra-person variability across the menstrual cycle. Throughout our analyses, we found that the rhythmic pattern of glucose variation persisted regardless of menstrual cycle duration.

Our results serve as a basis for conversations on the interpretation of glucose levels with respect to menstrual health^23^. Individuals who anticipate adjusting their behaviors (e.g., diet^24^ and sleep^25^) based on their glucose variation may need to consider how menstruation influences their interpretation of this data. For example, those who have higher glucose levels while experiencing stronger food cravings might contemplate different symptom management strategies depending on how far along they are in their menstrual cycle. Accounting for glucose variation across the menstrual cycle is also imperative for individuals with conditions like type 1 diabetes, as increased glucose levels during the luteal phase may lead to hyperglycemia in diabetic individuals and should be considered when planning insulin therapy^26^.

In addition, the biphasic pattern we found in daily median glucose levels across the menstrual cycle reveals an opportunity to examine it as a parameter for menstrual phase estimation. Other physiological parameters that have been suggested for menstrual phase prediction include pulse rate^27^, basal body temperature^28^, and cervical mucus^29^. As noted by Shilaih et al. ^27^, continuous physiological parameters might especially benefit individuals who experience varied menstrual cycle durations because conventional alternatives (e.g., self-reporting) may not adequately capture their menstrual experiences. Thus, we posit that blood glucose may also be a salient parameter to include in multimodal methods for monitoring menstrual health, such as for fertility awareness^27^ or menstrual cycle length prediction^30^.

Currently, identifying different physiological trends in menstrual contexts is challenging because collating and interpreting signals across devices is rarely supported^31^. To mediate this issue, researchers should consider developing visualizations and applications that integrate data into a single location. This goal may be difficult to achieve without sufficient interoperability being provided by device manufacturers. Even if interoperability is supported, people may not use all possible devices at the ideal frequency. An alternative method for supporting trend identification might be a singular device that collects a multitude of signals relevant to menstrual health. Devices such as smartwatches already cover a number of biomarkers (e.g., step count)^32^, but blood glucose and hormone levels are often notable exceptions. In the future, researchers might examine how glucose monitoring and hormone tracking could be included in such a form factor using innovative optical methods^33,34^.

There exist limitations in our study that future work should examine. Our study was limited by the narrow distribution we had in multiple demographic variables. Previous work has demonstrated that BMI, weight, and height are significant parameters for blood glucose variance across menstrual phases^4^; however, our results likely showed insignificant associations with respect to these variables due to their limited variation in our dataset.

Our explanatory variables could have also been made more reliable and quantitative. We used the Mira device to capture highly granular menstrual cycle data, and Mira claims to be at least as accurate as lab alternatives (e.g., 99% accuracy at predicting ovulation^35–37^); still, future research may consider validating our findings using clinical tests for determining menstrual cycle phase. In addition, we asked participants to self-report multiple facets of their daily experience for the sake of maximizing convenience and protocol adherence. Although there are more comprehensive ways of self-reporting some of these experiences, we limited our protocol to simpler reporting strategies to maintain high adherence. For example, we could have asked participants to maintain a journal to record the types and quantities of food that they ate. However, prior literature has noted that food journaling can be burdensome, difficult to sustain, and inaccurate, even with apps that provide a database of common foods to facilitate this practice^38^. Simply asking our participants to rate their appetite and food cravings helped us maximize the longitudinality and completeness of our other measures.

We also recognize that some of the self-reported experiences we collected could be replaced with more objective alternatives. For instance, sleep trackers like smartwatches and mattress sensors could be used to estimate sleep duration and detect arousals as a proxy for sleep quality. Examining health dimensions through other collection means more closely may reveal interesting associations worth further consideration.

## Methods

### Subjects

Fifty volunteer Canadians were recruited via social media groups and workspaces operated by women’s health advocacy organizations in the Greater Toronto Area. Recruitment was limited to non-diabetic, menstruating participants between 18 and 30 years old who did not intend on traveling outside of the Greater Toronto Area for significant durations of the study. Individuals were excluded if they were using hormonal therapy or hormonal contraception three months prior to or during the study. Participants were allowed to withdraw at any point of the study of their own volition and were de-enrolled if they became pregnant or failed to adhere to the study protocol.

### Study design and data collection

This study protocol was approved by the Research Ethics Board at the University of Toronto under Protocol #41568, and all participants provided informed consent electronically through e-signatures. Participants collected multimodal data at home over the course of three months, with the total study period being approximately seven months from the start date of the first participant to the end date of the last participant. Demographic data was collected at the start of the study with participants reporting their age (yrs), height (cm), and weight (kg) in an electronic survey.

All devices used in this study were commercially available and FDA-approved. Each participant wore a Dexcom G6 CGM^39^ to measure blood glucose and a Fitbit Sense smartwatch^40^ to measure step count. Participants were instructed to wear the CGM and smartwatch at all times during the study except during maintenance and charging. They also used Mira Plus Starter Kit^41^ urine tests to measure the levels of hormones relevant to the menstrual cycle, namely luteinizing hormone (LH) and estrone-3-glucuronide (E3G). Each kit included a hormone analyzer device, disposable urine test wands, and small cups to collect samples. Participants were instructed to complete the tests each morning after waking up, refraining from drinking liquids two hours prior to testing. After collecting a urine sample, participants dipped a wand into the cup for 15–20 s before inserting it into the analyzer device, which takes about 16 min to produce a measurement. Participants then recorded and sent these measurements to the researchers through a daily electronic survey.

Participants also kept an electronic diary to record the significance of multiple experiences relevant to their menstrual cycle. Fatigue, sleep issues, food cravings, and bloating were self-reported on the following scale: "Did not experience", "Very low", "Low", "Moderate", "High", and "Very high". Appetite and exercise levels were self-reported on the following scale: "Very low", "Low", "Moderate", "High", and "Very high". We note that although the Fitbit recorded participants’ step count, asking participants to self-report their exercise level provided complementary information about their physical activity. While step count was quantitative and objective, self-reported exercise level covered all possible forms of physical activity beyond what would be captured by a step counter (e.g., swimming, weightlifting). Similar to the hormonal urine tests, the electronic diaries were completed each morning. Participants were only allowed to submit complete diary entries in order to avoid additional data missingness.

### Data processing

Mira estimates users’ "fertile window" and menstruation days using a proprietary algorithm that identifies rises and falls in hormone data. We use this information to label four phases of the menstrual cycle: late-follicular (the last day of menstrual flow to the first day of the fertile window), ovulation (the fertile window), luteal (the last day of the fertile window to the first day of menstrual flow), and menstruation (days with menstrual flow). Given known inter-person variability in cycle length^42^, we also examined daily progression through the cycle as a percentage of the cycle length; for example, the fourth day of a 28-day cycle would be represented as 4/28 = 14%.

One participant dropped out of the study due to health issues. The remaining 49 participants collected data for an average of 79.3 ± 21.2 days for a total of 177 cycles and 640 cycle phases. To enhance the reliability of menstrual phase representations, we excluded any cycles with more than 4 consecutive days of missing hormone data (*N* = 3; 1.7%); this threshold corresponds to the shortest phase observed in our dataset. After excluding additional cycles where participants were missing data from at least 50% of the cycle days, 149 cycles and 554 phases remained. Each participant in this dataset logged an average of 3.1 ± 1.0 cycles, and these cycles lasted for an average of 28.0 ± 8.7 days.

Total step count and median glucose levels were calculated for each day (12:00 AM–11:59 PM) in order to consolidate continuous physiological data into daily values. To ensure that these summary statistics were representative of daily physiology, we verified whether participants wore the respective devices for at least 18 h within a given day. This was a trivial process for the Dexcom CGM data since the device reports glucose levels every 5 min. For the Fitbit, we established Fitbit usage by examining the amount of heart rate data that was recorded by the device since, unlike the other sensors, the heart rate sensor always reported new values every 5 s as long as the user was wearing the device. Within a given cycle, the average proportion of days that included sufficient Fitbit, Dexcom CGM, and diary data were 98.3 ± 0.6%, 86.8 ± 0.1%, and 90.3 ± 1.5%, respectively.

### Statistics

We report descriptive statistics for participant demographics, physiological signals, and self-reported experiences in aggregate and separated according to menstrual cycle phase. To examine if physiological signals and experiences varied across cycle phases, we conducted *χ*^2^ and repeated measures ANOVA tests. We first visualize daily median glucose, estrogen, and luteinizing hormone levels with LOESS smoothing across the menstrual cycle. We then ran a univariate model with a periodic restricted cubic spline to examine how daily blood glucose levels varied according to menstrual cycle phase progression. After that, we assessed the influence of menstruation, physiological data, demographics, and self-reported experiences on daily median glucose levels using linear mixed-effects models. We generated univariate models of all potential confounders, after which we generated a multivariate model with all confounders that had a statistically significant association with glucose levels in univariate models. To understand which confounders were influenced by menstrual cycle phase, we examined interactions between menstrual cycle phases and each confounder. To achieve this, we generated separate models for each significant confounder from the univariate model analysis with an interaction term between the respective confounder and menstrual cycle phase. Whenever significant interaction terms were identified, we stratified those analyses by splitting the dataset according to menstrual cycle phase and then generating separate univariate models for each dataset split.

The outcome variable of the aforementioned models was always daily median glucose level, while the other signals were used as explanatory variables. Categorical variables were included as factor variables using the 5 or 6 levels described above; the lowest level ("did not experience" or "very low") of each question was used as the reference category. Although selecting a single reference category for menstrual cycle phase can be difficult due to the cyclic nature of menstruation^43^, we chose to set the late-follicular phase as the reference as it was the phase with the lowest daily median glucose levels. All of the models used random slopes and intercepts to account for blood glucose variance between and within participants. We report the *β*-coefficients, 95% confidence intervals (CI), and significance of each relationship examined in these models. Statistical significance was set at *p* < 0.05. All analyses were conducted in R-3.5.1 using the lme4^44^, peRiodiCS^45^, tidyverse^46^, and sjPlot^47^ packages.

### Reporting summary

Further information on research design is available in the Nature Research Reporting Summary linked to this article.

### Supplementary information

Supplementary Information

Reporting Summary

## Data Availability

On reasonable request, the dataset generated during and/or analyzed in this paper is available from the corresponding author.

## Code availability

Data is collated in SQL databases and analyzed using R-3.5.1. The code used for the analysis of the dataset is available upon reasonable request from the corresponding author.
