# Supplementary material for: Blood glucose variance measured by continuous glucose monitors across the menstrual cycle
Source: NPJ Digit Med. 2023 Aug 11;6:140. doi: 10.1038/s41746-023-00884-x (PMC10421863; doi:10.1038/s41746-023-00884-x)
Supplement: Supplementary file 1 — Supplementary Information [file 41746_2023_884_MOESM1_ESM.pdf]

Supplementary Information

	F	χ^2	df	p-value
**Physiological Signals**				
Blood Glucose (mmol/L)	20.20		3	<0.001
Luteinizing Hormone (mIU/mL)	181.00		3	<0.001
Estrogen (ng/mL)	86.80		3	<0.001
Step Count	4.57		3	<0.01
**Demographics**				
Age (yrs)	2.31		3	0.07
Height (cm)	2.24		3	0.08
Weight (kg)	2.38		3	0.07
BMI (kg/m^2)	1.56		3	0.20
**Daily Self-reported Experiences**				
Appetite		5.20	12	0.82
Exercise Level		17.19	12	0.04
Food Cravings		36.47	15	0.002
Bloating		110.39	15	<0.001
Fatigue		27.52	15	0.02
Sleep Issues		29.29	15	0.01

Supplementary Table 1. Detailed results of the statistical tests conducted for Table 1, including F-statistics and p-values for the ANOVAs and χ^2 statistics and p-values for the χ^2 tests.

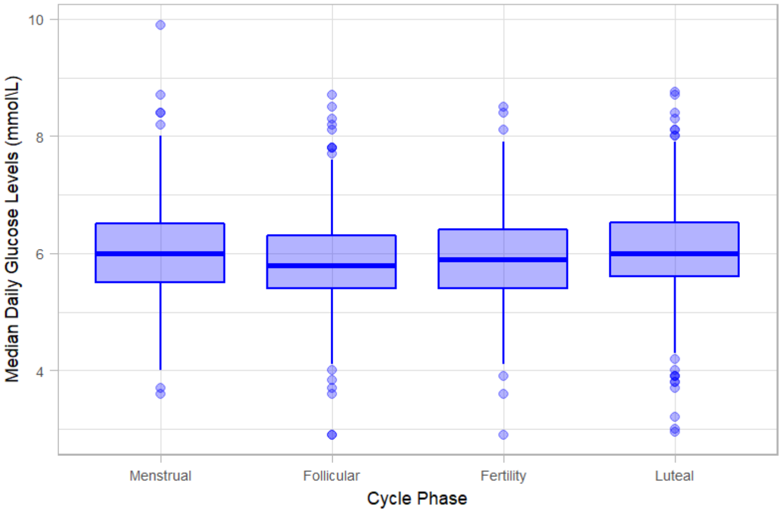

Supplementary Figure 1. Variation in raw median daily glucose levels across menstrual cycle phases. The center line represents the median, bounds of box represents the lower (Q1) and upper (Q3) quartiles, while the error bars represent the standard deviation.
