# Supplementary material for: Blood glucose variance measured by continuous glucose monitors across the menstrual cycle
Source: NPJ Digit Med. 2023 Aug 11;6:140. doi: 10.1038/s41746-023-00884-x (PMC10421863; doi:10.1038/s41746-023-00884-x)
Supplement: Supplementary file 2 — Reporting Summary [file 41746_2023_884_MOESM2_ESM.pdf]

nature portfolio

Corresponding author(s): Georgianna Lin

Last updated by author(s): Jul 7, 2023

Reporting Summary

Nature Portfolio wishes to improve the reproducibility of the work that we publish. This form provides structure for consistency and transparency in reporting. For further information on Nature Portfolio policies, see our Editorial Policies and the Editorial Policy Checklist.

Statistics

For all statistical analyses, confirm that the following items are present in the figure legend, table legend, main text, or Methods section.

n/a	Confirmed	
☐	☒	The exact sample size (n) for each experimental group/condition, given as a discrete number and unit of measurement
☐	☒	A statement on whether measurements were taken from distinct samples or whether the same sample was measured repeatedly
☐	☒	The statistical test(s) used AND whether they are one- or two-sided<br>*Only common tests should be described solely by name; describe more complex techniques in the Methods section.*
☐	☒	A description of all covariates tested
☒	☐	A description of any assumptions or corrections, such as tests of normality and adjustment for multiple comparisons
☐	☒	A full description of the statistical parameters including central tendency (e.g. means) or other basic estimates (e.g. regression coefficient) AND variation (e.g. standard deviation) or associated estimates of uncertainty (e.g. confidence intervals)
☐	☒	For null hypothesis testing, the test statistic (e.g. F, t, r) with confidence intervals, effect sizes, degrees of freedom and P value noted<br>*Give P values as exact values whenever suitable.*
☒	☐	For Bayesian analysis, information on the choice of priors and Markov chain Monte Carlo settings
☒	☐	For hierarchical and complex designs, identification of the appropriate level for tests and full reporting of outcomes
☒	☐	Estimates of effect sizes (e.g. Cohen's d, Pearson's r), indicating how they were calculated

Our web collection on statistics for biologists contains articles on many of the points above.

Software and code

Policy information about availability of computer code

Data collection	All devices used in this study to collect physiological data were commercially available and FDA-approved. Each participant wore a Dexcom G6 CGM to measure blood glucose and a Fitbit Sense smartwatch to measure step count. They also used Mira Plus Starter Kit urine tests to measure the levels of hormones relevant to the menstrual cycle, namely luteinizing hormone (LH) and estrone-3-glucuronide (E3G). Each kit included a hormone analyzer device, disposable urine test wands, and small cups to collect samples. All other study data, electronic diary and demographics forms, were collected through the survey platform Qualtrics using a link provided by research team.
Data analysis	All analyses were conducted in R-3.5.1 using the lme4, peRiodiCS, tidyverse, and sjPlotpackages.

For manuscripts utilizing custom algorithms or software that are central to the research but not yet described in published literature, software must be made available to editors and reviewers. We strongly encourage code deposition in a community repository (e.g. GitHub). See the Nature Portfolio guidelines for submitting code & software for further information.

Data

Policy information about availability of data

All manuscripts must include a data availability statement. This statement should provide the following information, where applicable:

- Accession codes, unique identifiers, or web links for publicly available datasets
- A description of any restrictions on data availability
- For clinical datasets or third party data, please ensure that the statement adheres to our policy

On reasonable request, the dataset used in this paper is available from the corresponding author.

Research involving human participants, their data, or biological material

Policy information about studies with human participants or human data. See also policy information about sex, gender (identity/presentation), and sexual orientation and race, ethnicity and racism.

Reporting on sex and gender	Our findings only apply to those who menstruate regardless of gender. We do not use sex and gender interchangeably in our analyses to acknowledge that those who menstruate may not all identify as the same gender. We did not conduct any sex and gender-based analyses as all participants shared the same sex. Additionally, all participants were not currently experiencing any form of hormonal therapy, thus their self-reported gender identity was unlikely to impact their physiological signals.
Reporting on race, ethnicity, or other socially relevant groupings	The only demographics we collected in our analyses were age (yrs), height (cm), and weight (kg). These variables have been shown in previous work to be confounders on blood glucose levels. We did not use any socially constructed categorization variables.
Population characteristics	The research sample contained young menstruating individuals in the Greater Toronto Area. Fifty volunteer Canadians were recruited via social media groups and workspaces operated by women's health advocacy organizations in the Greater Toronto Area. Recruitment was limited to non-diabetic, menstruating participants between 18 and 30 years old. We focused our efforts on recruiting participants under the age of 30 since people past perimenopause can have significantly different needs and expectations and should be examined separately with their transition status. Individuals were excluded if they were using hormonal therapy or hormonal contraception three months prior to or during the study as these methods alter an individuals' menstrual cycle physiology. The average age of our participants was around 20.8 years old. Our racial demographics reflected similar distributions as those in the Greater Toronto Area (e.g., 31% East Asian, 27% Caucasian, 18% South Asian, 9% Middle Eastern, 6% African, 9% Other).
Recruitment	Participants were recruited recruited through convenience and snowball sampling from social media groups and workspaces operated by women's health advocacy organizations in the Greater Toronto Area. The members of these groups tend to be more willing to participate in women's health studies and adhere to data collection requirements, but may not reflect the average menstrual literacy of an individual in the Greater Toronto Area. These biases are unlikely to affect our results as menstrual literacy is unlikely to directly impact an individual's physiological signals and menstrual experiences.
Ethics oversight	Our study was approved by the Research Ethics Board at the University of Toronto.

Note that full information on the approval of the study protocol must also be provided in the manuscript.

Field-specific reporting

Please select the one below that is the best fit for your research. If you are not sure, read the appropriate sections before making your selection.

☐ Life sciences ☒ Behavioural & social sciences ☐ Ecological, evolutionary & environmental sciences

For a reference copy of the document with all sections, see nature.com/documents/nr-reporting-summary-flat.pdf

Behavioural & social sciences study design

All studies must disclose on these points even when the disclosure is negative.

Study description	In this quantitative study, we used periodic restricted cubic splines to evaluate the relationship between blood glucose and the menstrual cycle, after which we assessed phase-based changes in daily median glucose level and associated physiological parameters using mixed-effects models.
Research sample	The research sample contained young menstruating individuals in the Greater Toronto Area. Fifty volunteer Canadians were recruited via social media groups and workspaces operated by women's health advocacy organizations in the Greater Toronto Area. Recruitment was limited to non-diabetic, menstruating participants between 18 and 30 years old. We focused our efforts on recruiting participants under the age of 30 since people past perimenopause can have significantly different needs and expectations and should be examined separately with their transition status. Individuals were excluded if they were using hormonal therapy or hormonal contraception three months prior to or during the study as these methods alter an individuals' menstrual cycle physiology.

	The average age of our participants was around 20.8 years old. Our racial demographics reflected similar distributions as those in the Greater Toronto Area (e.g., 31% East Asian, 27% Caucasian, 18% South Asian, 9% Middle Eastern, 6% African, 9% Other).
Sampling strategy	Participants were recruited recruited through convenience and snowball sampling. As no previous research was available on which to base standard deviation estimates, no sample-size calculation was calculated. We determined sample size based on sample sizes from previous work with similar data collection methods and length of time.
Data collection	All devices used in this study to collect physiological data were commercially available and FDA-approved. Each participant wore a Dexcom G6 CGM to measure blood glucose and a Fitbit Sense smartwatch to measure step count. They also used Mira Plus Starter Kit urine tests to measure the levels of hormones relevant to the menstrual cycle, namely luteinizing hormone (LH) and estrone-3-glucuronide (E3G). Each kit included a hormone analyzer device, disposable urine test wands, and small cups to collect samples. All other study data, electronic diary and demographics forms, were collected through the survey platform Qualtrics using a link provided by research team. Participants collected all data without researcher presence in their own homes.
Timing	The start date of data collection was January 9th, 2022 to June 11th, 2022. Participants entered the study on a rolling basis. Every participant collected around 90 days of data. The study ended when the final participant finished their three month data collection period.
Data exclusions	Data was excluded from analyses if they were determined incomplete. Data included values that were calculated for each day (12:00 AM–11:59 PM) in order to consolidate continuous physiological data and electronic diary responses into daily values. Different incompletion criteria per collection method was set before the study. For example, to enhance the reliability of menstrual phase representations, we excluded any cycles with more than 4 consecutive days of missing hormone data (N = 3; 1.7%); this threshold corresponds to the shortest phase observed in our dataset. After excluding additional cycles where participants were missing data from at least 50% of the cycle days, 149 cycles and 554 phases remained of the original total of 177 cycles and 640 cycle phases. For the other collection methods, 18 hours of Fitbit wear time was required for step count; 18 hours of Dexcom wear time was required for daily median glucose value; and a fully complete electronic diary entry was required for the self report menstrual experience values to be included in analyses for one particular day. Within a given cycle, the average proportion of days that then included sufficient Fitbit, Dexcom CGM, and diary data were 98.3%, 86.8%, and 90.3% respectively.
Non-participation	Only one participant dropped out of the study citing health issues that may impact data collection adherence.
Randomization	Participants were not allotted into experimental groups.

Reporting for specific materials, systems and methods

We require information from authors about some types of materials, experimental systems and methods used in many studies. Here, indicate whether each material, system or method listed is relevant to your study. If you are not sure if a list item applies to your research, read the appropriate section before selecting a response.

Materials & experimental systems

n/a	Involved in the study	
☒	☐	Antibodies
☒	☐	Eukaryotic cell lines
☒	☐	Palaeontology and archaeology
☒	☐	Animals and other organisms
☒	☐	Clinical data
☒	☐	Dual use research of concern
☒	☐	Plants

Methods

n/a	Involved in the study	
☒	☐	ChIP-seq
☒	☐	Flow cytometry
☒	☐	MRI-based neuroimaging
